# Supplementary material for: How many online workers are there in the world? A data-driven assessment
Source: Open Res Eur. 2021 Oct 15;1:53. Originally published 2021 May 19. [Version 4] doi: 10.12688/openreseurope.13639.4 (PMC10445891; doi:10.12688/openreseurope.13639.4)
Supplement: Peer review history

# Peer review: Reviewer response for version 3

**DOI:** 10.21956/openreseurope.15305.r27768

**Published:** 2021-10-07

**Authors:** Richard Heeks

**Affiliations:** 1 Global Development Institute, University of Manchester, Manchester, UK

Issues identified in the previous review have all been addressed.

If applicable, is the statistical analysis and its interpretation appropriate?

Partly

Is the study design appropriate and is the work technically sound?

Yes

Is the work clearly and accurately presented and does it engage with the current literature?

Partly

Are the conclusions drawn adequately supported by the results?

Yes

Are sufficient details of methods and analysis provided to allow replication by others?

Yes

Are all the source data and materials underlying the results available?

Yes

Reviewer Expertise:

Digital economy, digital development

I confirm that I have read this submission and believe that I have an appropriate level of expertise to confirm that it is of an acceptable scientific standard.

# Peer review: Reviewer response for version 2

**DOI:** 10.21956/openreseurope.15178.r27512

**Published:** 2021-09-24

**Authors:** Annarosa Pesole

**Affiliations:** 1 European Commission - Joint Research Centre, Institute for Prospective Technological Studies (IPTS), Seville, Spain

Dear authors, thanks for your efforts.

 Here some comments:

*“ Figure 2 summarises the performance of the predictive model graphically. As is expected, the correlation between observed and predicted numbers of registered workers is exactly 1.”*

 Indeed, I would have been surprised if correlation was different from 1. What I wanted to suggest in my previous review, clearly I didn’t express it well, it was (if possible) to use the number of registered users of (some or one) the platform for which authors also know the number of workers, impute the number of workers in the selected platform to zero and then check if the estimate number was close to the observed one. Perhaps such a test cannot be performed as I am imagining that it will be impossible to isolate one single platform and also that changing the validation set will modify the algorithmic process. I still believe that the paper could benefit from some more practical intuitions about strength and limitations of the methodology.

 Some footnotes are misplaced

If applicable, is the statistical analysis and its interpretation appropriate?

Partly

Is the study design appropriate and is the work technically sound?

Partly

Is the work clearly and accurately presented and does it engage with the current literature?

Yes

Are the conclusions drawn adequately supported by the results?

Partly

Are sufficient details of methods and analysis provided to allow replication by others?

Yes

Are all the source data and materials underlying the results available?

Yes

Reviewer Expertise:

Labour economics, digital labour platforms.

I confirm that I have read this submission and believe that I have an appropriate level of expertise to confirm that it is of an acceptable scientific standard.

# Peer review: Reviewer response for version 2

**DOI:** 10.21956/openreseurope.15178.r27514

**Published:** 2021-09-02

**Authors:** Richard Heeks

**Affiliations:** 1 Global Development Institute, University of Manchester, Manchester, UK

The paper has been improved but there continue to be errors:

- Despite the author response statement to have amended this (“We now clarified that we refer to “those who completed at least 10 projects OR earned at least US$1,000 in the last 30 days””), there is still an ongoing lack of clarity about the third measure of workers.  In the abstract and in the first and second appearance of this criterion in the text in the Methods section and also in Table 1 and Table 2 still lacks the “.. in the last 30 days” addition; then in the ‘Inferring the number workers who have worked’ section the measure is listed as “have worked over the past 30 days”; then in the results it’s back to “total earnings of over $1000 or over 10 completed projects”.  This is inconsistent generally and also specifically (‘at least 10’ is not the same as ‘over 10’, ditto with the $ figure).
- More generally, refer to earlier comments about the third measure e.g. incorrect assumption of equivalence to full-time work.
- Methods section, should be “.. each platform: number of registered worker profiles ..”
- Footnote 5 is misnumbered in the text.
- Heading should be “Inferring the number of workers who have worked”.
- Results: how do you get from 9.3m and 2.2m to 75m and 21m.  Explain and justify.

If applicable, is the statistical analysis and its interpretation appropriate?

Partly

Is the study design appropriate and is the work technically sound?

Yes

Is the work clearly and accurately presented and does it engage with the current literature?

Partly

Are the conclusions drawn adequately supported by the results?

Yes

Are sufficient details of methods and analysis provided to allow replication by others?

Yes

Are all the source data and materials underlying the results available?

Yes

Reviewer Expertise:

Digital economy, digital development

I confirm that I have read this submission and believe that I have an appropriate level of expertise to confirm that it is of an acceptable scientific standard, however I have significant reservations, as outlined above.

# Response

**Published:** 2021-10-04

**Authors:** Fabian Stephany

**Affiliations:** 22137, UK

Dear reviewer, thank you for your time and consideration in reading our revised manuscript and in suggesting further improvements.

 We have made the following edits based on your valuable suggestions:

 The third group of workers - after registered workers and workers, who have worked at least once - is now called workers, who have worked significantly. This means that these workers have, at some point, gained a significant part of their income from working on the platform. We avoid the terminology of "full-time" workers, as we can not say with certainty that these workers are currently engaged in "full-time" acitivities on the platform. Workers in this third category have either completed AT LEAST 10 projects OR have AT LEAST earned 1000 USD in the past. This definition DOES NOT include any reference to a 30 day timespan. The text has been adapted with regard to this definition.

 Our Results Section concludes "...that there could at most be as many as 205 million registered worker profiles, 75 million workers who have ever worked through an online labour platform, and 21 million workers, who have worked significantly". These numbers result from multiplying the upper benchmark of the number of registered workers (205 million) with the respective upper confidence intervals for workers who have ever worked (36.8%) and workers, who have worked significantly (10%).

 Spelling and footnote setting errors have been corrected. 

 Thanks again for your contributions and time. Best,  The authors

# Peer review: Reviewer response for version 1

**DOI:** 10.21956/openreseurope.14710.r26940

**Published:** 2021-07-16

**Authors:** Valeria Cirillo

**Affiliations:** 1 Department of Political Science, University of Bari Aldo Moro, Bari, Italy

The paper deals with an interesting and crucial topic that is the quantification of platform workers over the world. Although platforms rely on an increasing number of workers (as aggregate statistics have shown with respect to food delivery sector during the Covid crisis), a punctual estimation of the size of platform economy is still missing. Therefore, combining data from several sources, the authors follow a two-step strategy to provide a first assessment of the number of online workers globally. In this regard, the paper is ambitious and represents one of the first attempt to provide an absolute value of individuals offering their workforce through an online digital platform. Furthermore, while many contributions focus on gig workers – that is the fraction of workers working for platforms operating in local labour markets and therefore offering tangible services in exchange of money -, the authors decide to map the large population of online freelancers often neglected by official statistics.

 The paper is short and well-focused. It offers new insights on the quantification of platform economy by adopting a data-driven approach to predict the number of workers for the platforms for which this information is not available. The novelty of the approach is to collect a list of publicly available predictive features and to train a machine learning model to predict the number of workers for the platforms where this information is not available. This is a very interesting application of machine learning algorithms which deserve to be detailed in the manuscript.

 The two-step strategy is crucial for the development of the paper and should be better described. The first step entails the compilation of a complete census of all online labour platforms by relying on: (i) Crunchbase; (ii) cross-regional survey in Wood et al. (2019); (iii) Google search of specific verbal locutions. Can we know which platforms have been selected? I think it is interesting to focus on online freelance economy, however the reader would like to acquire more info about these 351 platforms included. Can these platforms be grouped according to the prevailing type of activity? I suggest emphasizing this part of the research showing the main outputs.

 Furthermore, the authors should provide more details on the so-called known population, that is the fraction of platforms for which information of workers is available. If platforms are grouped according to the prevailing type of activity (for example, platforms specialized in high-skilled consultancies, middle-skill or low-skill, all type of skills), some descriptive features can be traced.

 There are few typos in the text that need to be corrected.

 Overall, I think this is a nice paper, very useful, that can pave the way to further research aiming to quantify the size of platform work. Related to this point, few sentences in the conclusion can be added to illustrate next steps in the research.

If applicable, is the statistical analysis and its interpretation appropriate?

Yes

Is the study design appropriate and is the work technically sound?

Yes

Is the work clearly and accurately presented and does it engage with the current literature?

Yes

Are the conclusions drawn adequately supported by the results?

Yes

Are sufficient details of methods and analysis provided to allow replication by others?

Partly

Are all the source data and materials underlying the results available?

Yes

Reviewer Expertise:

Labour Market and Technology; Inequalities; Occupational dynamics; Wages

I confirm that I have read this submission and believe that I have an appropriate level of expertise to confirm that it is of an acceptable scientific standard.

# Response

**Published:** 2021-08-25

**Authors:** Fabian Stephany

**Affiliations:** 22137, UK

Dear reviewer,

 Thank your very much for your time and effort - the revised version of this work has benefited significantly from your comments. In summary, based on the suggestions made by all reviewers, we have corrected some calculation errors, made further clarifications on the rationale and procedure of our big data approach, and provided additional graphical evidence for the goodness of our estimations. In addition to the supplementary material of the initial version, we now provide a summary table listing all 351 platforms that are included in our analysis together with information on the type of online work performed on the platform, the number of observed (experienced) workers, and details on where this information has been retrieved.

 In the following, we would like to respond to your individual comments:

*“The two-step strategy is crucial for the development of the paper and should be better described. The first step entails the compilation of a complete census of all online labour platforms by relying on: (i) Crunchbase; (ii) cross-regional survey in Wood et al. (2019); (iii) Google search of specific verbal locutions. Can we know which platforms have been selected? I think it is interesting to focus on online freelance economy, however the reader would like to acquire more info about these 351 platforms included. Can these platforms be grouped according to the prevailing type of activity? I suggest emphasizing this part of the research showing the main outputs.”*

 This is an important suggestion. A new table, which is provided in the data repository linked to the paper, now provides this information on all 351 platforms.  

*“Furthermore, the authors should provide more details on the so-called known population, that is the fraction of platforms for which information of workers is available. If platforms are grouped according to the prevailing type of activity (for example, platforms specialized in high-skilled consultancies, middle-skill or low-skill, all type of skills), some descriptive features can be traced.”*

 This information is also provided in the new table in the data repository.

# Peer review: Reviewer response for version 1

**DOI:** 10.21956/openreseurope.14710.r26937

**Published:** 2021-06-21

**Authors:** Richard Heeks

**Affiliations:** 1 Global Development Institute, University of Manchester, Manchester, UK

Gig work – short-term tasks mediated by digital platforms – is a topic of growing research, policy, and practice interest. However, its novelty, existence largely outside conventional systems of labour statistics, and the commercial confidentiality of the gig platforms themselves mean that we are often fumbling around in the dark to understand two key things: how big is this sector, and how fast is it growing (assuming that it is growing). Estimation is also hampered by definitional variance, with broader definitions coming close to those for all types of self-employment – for example, estimating that 36% of the US workforce are gig workers (McCue 2018).

 This paper is therefore a very welcome addition given it attempts a better approach to estimation than we have seen previously; albeit restricted to online gig work and thus excluding those engaged with physical gig work such as seen for platforms such as Uber, Didi, Gojek, etc. The paper is relatively brief and well-focused: while situating its findings it does not drift into discussion of broader issues but keeps to the track of its core objective, to “produce an estimate of the total number of online freelancers globally”.

 The critique of prior estimates is valuable; for example, helping steer future research away from limitations of past work. The methods used are explicit and for the compilation of platforms and also for the observation and prediction, look robust enough. The researchers deal well and openly with the uncertainties of their approach, and provide some valuable pointers to key issues such as ratio of registered to active workers. Unfortunately, there seem to be some basic errors in figures indicated or calculated in the text of the current version; errors that need to be rectified.

 It is valuable that the authors provide access to their dataset. There are a number of ways in which the paper could fairly quickly and easily provide additional value:

- I wonder if there is any logic in putting the list of platform names onto a wiki-like site that others could add to or at least comment on.
- Likewise, this could be derived from the dataset but it would be useful for readers to have a table of, say, the top 20 observed platforms by size – e.g. those in the 1m+ categories, and also an Appendix table with all 162 platforms, their type and worker nos. That could significantly increase the value and utilisation (including citation) of the paper.
- On p4, it would be helpful to name the seven and six platforms with more accessible data: researchers will find that useful.

Amendments to consider:

- p3: “data disclosed by online labour” – is that meant to be “data disclosed by online labour platforms”?
- p4: the label for Figure 1 (45 out of 151) doesn’t seem to match the text – I suspect this may derive from an earlier draft of the paper.
- p5: what are the ‘Number of Workers’ figures in Table 1. These seem to relate to just the 162 platforms where data could be accessed.  If so, it seems a little unusual to mix that data in with data from the quite different methodology of the machine learning estimates. Wouldn’t the bottom half of Table 1 better be separated out and placed with the p4 material? Second, on p4 the authors state that Freelancer is one of the platforms covered by ‘ever worked’ and ‘worked last month’ – how does that fit with the max values for these being a few tens of thousands?
- p6: maybe just explain where 159 appeared from – it is neither the difference between 162 and 351, nor the difference between 162 and (Table 1) 339.
- Figures 3 and 4: I read the text and looked at the figures several times but could not understand them.  First, the x-axis – is 0.2 for example equivalent to 20% or 0.2%?  Why not just use percentages. Second, what does the y-axis show: share of what? Is there also a bit of a mismatch between the discontinuous categories for the three columns, and then plotting a mean as for a continuous variable? If the red line on Figure 3 is meant to represent 5% how does that fit with it being roughly half way between “0.0” and “0.2”. This could all be my ignorance but I suspect these two figures and their text explanation need reworking.  It later looks like the statistic should be 11% not 5%.
- p4-p7: what is the third measure. On p4 it is those “who completed at least 10 projects, or earned at least $1000”; on p6 it is those who “have worked over the past 30 days”; and it is also referred to as being equivalent to having a full-time job. It looks like clarification is required of what the actual measure is. In addition, calling either measure as akin to a full-time job seems inappropriate unless the measure is actually “those who completed at least 10 projects or earned at least US$1,000 in the last 30 days”.
- p7: “because they our results” should be “because our results”.
- p7: unclear how you get from 163 million to 19 million and 5 million.  p6 states: “On average, only 5% of registered users have ever worked, and 4% have worked over the past 30 days”.  19 million is around 12% of 163 million; 5 million is around 3% of 163 million. Some revision or at least explanation is required.  (Even if I try using the 1.83 divider and 1.21 multiplier, neither of these figures comes out and in any case and as stated on p8 these figures were not used here.) When you correct the figures on p6, I suggest you give to one decimal place, not zero decimal places.
- p8: again it looks like some basic maths has gone awry. If you multiply a number by 1.21, you do not get a smaller number!
- p8: first, it would be useful to show what the error bands are and how they were calculated.  Second, why not give both the lower as well as the higher error band estimate. As with other calculations, the figures may need to be redone given the issues identified with the main calculations.
- p8: would be useful to just be quite explicit about the ‘growing rapidly’ point that you mean compared to the 2015 figures from Kuek et al.
- p9: you have exceeded your quota of use of the word ‘nonetheless’: suggest you substitute for something else in one or two places.

If applicable, is the statistical analysis and its interpretation appropriate?

Partly

Is the study design appropriate and is the work technically sound?

Yes

Is the work clearly and accurately presented and does it engage with the current literature?

Partly

Are the conclusions drawn adequately supported by the results?

Yes

Are sufficient details of methods and analysis provided to allow replication by others?

Yes

Are all the source data and materials underlying the results available?

Yes

Reviewer Expertise:

Digital economy, digital development

I confirm that I have read this submission and believe that I have an appropriate level of expertise to confirm that it is of an acceptable scientific standard, however I have significant reservations, as outlined above.

## References

[1] : 57 Million U.S. Workers Are Part Of The Gig Economy. *Forbes* .2018; Reference source

# Response

**Published:** 2021-08-25

**Authors:** Fabian Stephany

**Affiliations:** 22137, UK

Dear reviewer,

 Thank you very much for your time and effort - the revised version of this work has benefited significantly from your comments. In summary, based on the suggestions made by all reviewers, we have corrected some calculation errors, made further clarifications on the rationale and procedure of our big data approach, and provided additional graphical evidence for the goodness of our estimations. In addition to the supplementary material of the initial version, we now provide a summary table listing all 351 platforms that are included in our analysis together with information on the type of online work performed on the platform, the number of observed (experienced) workers, and details on where this information has been retrieved.  

 In the following, we would like to respond to your individual comments:

*“I wonder if there is any logic in putting the list of platform names onto a wiki-like site that others could add to or at least comment on…..Likewise, this could be derived from the dataset but it would be useful for readers to have a table of, say, the top 20 observed platforms by size – e.g. those in the 1m+ categories, and also an Appendix table with all 162 platforms, their type and worker nos. That could significantly increase the value and utilisation (including citation) of the paper…..On p4, it would be helpful to name the seven and six platforms with more accessible data: researchers will find that useful.”*  

 A new table, which is provided in the data repository linked to the paper, now provides this information on all 351 platforms.

*“p3: “data disclosed by online labour” – is that meant to be “data disclosed by online labour platforms”?”*

 Yes, this has now been corrected.

*“p4: the label for Figure 1 (45 out of 151) doesn’t seem to match the text – I suspect this may derive from an earlier draft of the paper.”*  

 Indeed, this mistake has now been corrected.

*“p5: what are the ‘Number of Workers’ figures in Table 1. These seem to relate to just the 162 platforms where data could be accessed.  If so, it seems a little unusual to mix that data in with data from the quite different methodology of the machine learning estimates. Wouldn’t the bottom half of Table 1 better be separated out and placed with the p4 material? Second, on p4 the authors state that Freelancer is one of the platforms covered by ‘ever worked’ and ‘worked last month’ – how does that fit with the max values for these being a few tens of thousands?”*  

 Thanks for addressing these relevant issues. Table 1 contains only information on observed and not on estimated data. This has now been made explicit in the text. Furthermore, the data on the row “ever worked” has been corrected accordingly - we show the share of workers in the last two rows, illustrating the skewness of the data. In addition, a new table, which is provided in the data repository linked to the paper, now provides this information on all 351 platforms.

*“p6: maybe just explain where 159 appeared from – it is neither the difference between 162 and 351, nor the difference between 162 and (Table 1) 339.”*

 The number 159 results from 162 (observed platforms) minus the three Chinese platforms 680.com, epwk.com and zbj.com, as we are sceptic that Google trends and Alexa are capturing site popularity in China well, and the Poisson XGBoost model performed much better if these three observations were excluded. This reasoning is now summarised in a footnote.

*“Figures 3 and 4: I read the text and looked at the figures several times but could not understand them.  First, the x-axis – is 0.2 for example equivalent to 20% or 0.2%?  Why not just use percentages. Second, what does the y-axis show: share of what? Is there also a bit of a mismatch between the discontinuous categories for the three columns, and then plotting a mean as for a continuous variable? If the red line on Figure 3 is meant to represent 5% how does that fit with it being roughly half way between “0.0” and “0.2”. This could all be my ignorance but I suspect these two figures and their text explanation need reworking.  It later looks like the statistic should be 11% not 5%.”*

  This is a good suggestion. The numbers are now mentioned in the text and the graphs have been excluded. We show the share of workers in the last two rows of Table 1, illustrating the skewness of the data.

*“p4-p7: what is the third measure. On p4 it is those “who completed at least 10 projects, or earned at least $1000”; on p6 it is those who “have worked over the past 30 days”; and it is also referred to as being equivalent to having a full-time job. It looks like clarification is required of what the actual measure is. In addition, calling either measure as akin to a full-time job seems inappropriate unless the measure is actually “those who completed at least 10 projects or earned at least US$1,000 in the last 30 days”.”*

 We now clarified that we refer to “those who completed at least 10 projects OR earned at least US$1,000 in the last 30 days”. 

*“*

*p7: “because they our results” should be “because our results”.”*

 This has been corrected.

*“p7: unclear how you get from 163 million to 19 million and 5 million.  p6 states: “On average, only 5% of registered users have ever worked, and 4% have worked over the past 30 days”.* *19 million is around 12% of 163 million; 5 million is around 3% of 163 million. Some revision or at least explanation is required.  (Even if I try using the 1.83 divider and 1.21 multiplier, neither of these figures comes out and in any case and as stated on p8 these figures were not used here.) When you correct the figures on p6, I suggest you give to one decimal place, not zero decimal places.”*

 Thanks for pointing this out. Our calculations have been adjusted accordingly. We estimate: 163 million registered workers of which 11.6% (18.91 or 19 million) are active and 3% (4.89 or 5 million) completed at least 10 projects OR earned at least US$1,000 in the last 30 days.

*“p8: again it looks like some basic maths has gone awry. If you multiply a number by 1.21, you do not get a smaller number!”*

 In addition, we adjusted estimates for multi-homing (average worker is active on 1.83 platforms) and multi-working (21% of all workers do this). The number adjust accordingly, for active workers: 19  / 1.81 * 1.21 = 12.7 million and for  “full-time” workers: 5 / 1.81 * 1.21 = 3.34 million.

*“p8: would be useful to just be quite explicit about the ‘growing rapidly’ point that you mean compared to the 2015 figures from Kuek et al.”*

 We made this more explicit in the new version.

*“p9: you have exceeded your quota of use of the word ‘nonetheless’: suggest you substitute for something else in one or two places.”*

 Thanks, this has been adjusted.

# Peer review: Reviewer response for version 1

**DOI:** 10.21956/openreseurope.14710.r26939

**Published:** 2021-06-10

**Authors:** Annarosa Pesole

**Affiliations:** 1 European Commission - Joint Research Centre, Institute for Prospective Technological Studies (IPTS), Seville, Spain

Despite the growing interest in platform work, as of today, there is still little information about how spread the phenomenon is. This paper partially addresses this issue looking at the freelance activities mediated by digital platforms. The paper proposes an innovative data-driven methodology to assess the number of online freelancers building on a restricted number of platforms for which information are publicly available. The proposal for this new methodology stems from the current limitations of official labour statistics in depicting platform work. As correctly stated by the authors, the lack of a common definition together with the fragmented nature of this type of work make it very difficult for traditional statistical surveys to capture the prevalence of platform work.

 The authors start from compiling a list of online freelancing platforms gathering a total of 351 platforms, out of which 162 reported information about registered users and only 7 about workers who actually completed at least one task. In order to estimate the number of registered workers for those platforms that do not report the information they implement a creative solution using public data on website popularity combining information from Google trends and alexa rank.

 The idea is surely captivating and shows potential for the use of big data in traditional statistics. However, the high volume and volatility of stream data may bring noise accumulation and spurious correlation, creating issues in computational feasibility and algorithmic stability (Wang *et al*.2016) ^1^. Perhaps these limitations should have been better addressed in the paper and also the strategy to cope with these potential issues explained in greater details Following this procedure, the authors estimated a number of 23 million registered workers. A potential test for the goodness of the estimate could have been to repeat the same methodology for the platforms they have information and check if the predicted numbers were close to the reported ones.

 As they correctly point out, not all the users who register in the platform become workers (i.e. complete at least one task). From the platforms where information is available, they compute that on average only 5% of registered users have ever worked, however the estimated parameter for workers who completed one project is 11.6% (Table 2). A valuable information to help the readers understanding better it would have been to present a table with the real data for the 7 platforms with the full set of data, so to compare the magnitude and the real share of workers against the predicted one (i.e. if between the 7 platforms were to be included the 3 outliers, almost half of the sample (77 million) should have been multiplied by 5%).

 Multi-homing and multi-working are also taken into account highlighting the additional difficulties of correctly quantifying the number of workers in online freelancing platforms. In the result section, estimates adjusted for multi-homing and multi-working are reported, although the latter appear to be wrong as they should be 12 million and 3.2 million (as reported in page 7 “we can adjust our numbers for multiple workers working under a single account by multiplying the number of workers by 1.21.”, that is 10 by 1.21 and 2.7 by 1.21)

 The paper could benefit from: 1) adding a table with detailed information on the 7 platforms for which it was possible to observe workers. In particular specifying also how the information was retrieved. 2) Testing the goodness of the methodology replicating the data-driven approach for the platforms reporting the number of registered workers and comparing the estimated and the observed numbers.

 Despite data limitation and the need for more clarity in some methodological steps, the paper remains an interesting study bringing valuable insights on the debate about measurement. The novelty of the approach paves the way to a new stream of research stressing the importance and the challenges associated with the use of big data. I appreciate the efforts done by the authors and my overall comments are positive.

If applicable, is the statistical analysis and its interpretation appropriate?

Partly

Is the study design appropriate and is the work technically sound?

Partly

Is the work clearly and accurately presented and does it engage with the current literature?

Yes

Are the conclusions drawn adequately supported by the results?

Partly

Are sufficient details of methods and analysis provided to allow replication by others?

Yes

Are all the source data and materials underlying the results available?

Yes

Reviewer Expertise:

Labour economics, digital labour platforms.

I confirm that I have read this submission and believe that I have an appropriate level of expertise to confirm that it is of an acceptable scientific standard, however I have significant reservations, as outlined above.

## References

[1] : Statistical methods and computing for big data. *Stat Interface* .2016;9(4) : 10.4310/SII.2016.v9.n4.a1 399-414 10.4310/SII.2016.v9.n4.a1 27695593PMC5041595

# Response

**Published:** 2021-08-25

**Authors:** Fabian Stephany

**Affiliations:** 22137, UK

Dear reviewer,

 Thank you very much for your time and effort - the revised version of this work has benefited significantly from your comments. In summary, based on the suggestions made by all reviewers, we have corrected some calculation errors, made further clarifications on the rationale and procedure of our big data approach, and provided additional graphical evidence for the goodness of our estimations. In addition to the supplementary material of the initial version, we now provide a summary table listing all 351 platforms that are included in our analysis together with information on the type of online work performed on the platform, the number of observed (experienced) workers, and details on where this information has been retrieved. In the following, we would like to respond to your individual comments:

*“The idea is surely captivating and shows potential for the use of big data in traditional statistics. However, the high volume and volatility of stream data may bring noise accumulation and spurious correlation, creating issues in computational feasibility and algorithmic stability (Wang et al.2016) 1. Perhaps these limitations should have been better addressed in the paper and also the strategy to cope with these potential issues explained in greater detail. Following this procedure, the authors estimated a number of 23 million registered workers. A potential test for the goodness of the estimate could have been to repeat the same methodology for the platforms they have information and check if the predicted numbers were close to the reported ones.”*

 There is a new Figure 2 and explanation, illustrating this test for the goodness of the estimate.

*“As they correctly point out, not all the users who register in the platform become workers (i.e. complete at least one task). From the platforms where information is available, they compute that on average only 5% of registered users have ever worked, however the estimated parameter for workers who completed one project is 11.6% (Table 2). A valuable information to help the readers understanding better it would have been to present a table with the real data for the 7 platforms with the full set of data, so to compare the magnitude and the real share of workers against the predicted one (i.e. if between the 7 platforms were to be included the 3 outliers, almost half of the sample (77 million) should have been multiplied by 5%).”   -> A new table, which is provided in the data repository linked to the paper, now provides information on all 351 platforms: Only one of the three mentioned outliers, freelancer.com with 31 million workers (50877 experienced workers), is contained in the sample of 7 observed platforms.   “Multi-homing and multi-working are also taken into account highlighting the additional difficulties of correctly quantifying the number of workers in online freelancing platforms. In the result section, estimates adjusted for multihoming and multi-working are reported, although the latter appear to be wrong as they should be 12 million and 3.2 million (as reported in page 7 “we can adjust our numbers for multiple workers working under a single account by multiplying the number of workers by 1.21.”, that is 10 by 1.21 and 2.7 by 1.21).”*

 This mistake has now been corrected.

# Peer review: Reviewer response for version 1

**DOI:** 10.21956/openreseurope.14710.r26938

**Published:** 2021-06-07

**Authors:** Paola Tubaro

**Affiliations:** 1 Centre National de la Recherche Scientifique (CNRS), Université Paris-Saclay, Paris, France

This is a really valuable paper on an important subject. As the authors say, official statistical instruments (like Labour Force Surveys) fail to capture platform-based activities that challenge established definitions of salaried employment. Particularly difficult to measure is the little visible and geographically spread activity of online workers whose activities are delivered and paid remotely over the internet. The authors endeavour to fill precisely this gap, and produce an estimate of the total number of online freelancers globally, using publicly available data gathered from internet sources. The work they propose is highly innovative and relevant, and I will highlight three main strengths.

 First, while the authors (rightly) insist on the state-of-the-art machine learning techniques they leverage to estimate the number of workers on platforms that do not release this information, their work is also, to an extent, qualitative and mixed-methods. The very first step of their analysis, before any calculations, consisted in establishing a list of over 300 relevant online platforms, and gathering publicly available information about them (whether self-disclosed by the platforms or presented in other sources, e.g. the press or Wikipedia). This was done, basically, by hand, following a protocol that relies on an external, rich source (Crunchbase), a previous study of the same team, and a systematic online search. This should perhaps be recognized more explicitly, not only to give credit to the painstaking work of compiling the list, but also to discuss the issues that may have arisen – and that may add to the authors’ discussion of the uncertainties related to the calculation. Surely the authors had to make exclusion/inclusion decisions especially in ambiguous cases, such as platforms offering both local gigs and remote tasks (like Bemyeye), or platforms operating rather as traditional BPO firms (like Playment). A discussion of these issues as is often done in qualitative research (such as decision by consensus in the team, or majority choice…), may be helpful.

 Similarly, the authors must have had to make decisions on how to deal with heterogeneity of information released by platforms. As they notice, some platforms do not disclose any information at all, while others just provide number of registrations, not levels of engagement and activity. But even when they disclose information, platforms do not do so uniformly. Some give just orders of magnitude (such as Clickworker.com, 2200k workers in 2020) and update them very irregularly (such as Amazon, whose latest figures for Mechanical Turk date back to the early 2010s), others provide very precise numbers (as Microworkers.com which discloses its up-to-date numbers daily on its home page). Again, researchers have to make choices – take whatever platforms provide, approximations, or as precise figures as possible? And what happens when sources disagree?

 These issues illustrate well the ‘costs’, so to speak, of relying on disparate online sources, which unlike surveys, do not allow relying on shared definitions and/or prior knowledge of sampling distributions. Yet this is the only possible solution when surveys cannot help, and has the merit of using publicly-available information that allows for check and replication. In the data economy that thrives through platforms, one part of the world of work escapes established definitions and misses the gaze of official statistics. This is why we need to devise creative ways of observing the less-and-less observable.

 Another merit of the paper is to distinguish between registrations and actual levels of engagement. One may register on a platform for mere curiosity or to explore it as a journalist or as a researcher – as many of us indeed do. Additionally, some platforms such as Microworkers.com do not differentiate the two sides of the market, so that the total number of their registered users includes both workers and their clients/employers. Hence, the authors are right to count separately the users who worked at least once, and to further single out those who did at least 10 assignments or earned more than $1000. Indeed platform labour is not always an individual’s main activity and may constitute just a side hustle, or an occasional buffer in periods of unemployment.

 While this diversity of levels of engagement is widely recognized in the nascent literature on digital platform labour, it is not always operationalized in the same way. For example, Gray and Suri (2019, p. 104) ^1^ distinguish three groups, the ‘experimentalists’, ‘regulars’, and ‘always-on’, while Urzi-Brancati *et al.* (2020, p. 15) ^2^ propose a four-level classification with ‘sporadic’, ‘marginal’, ‘secondary’ and ‘main’ platform workers. Admittedly, a more homogeneous approach is out of reach because these studies differ in scope (as the latter, for example, also includes location-based platform workers) and types of available data. Nevertheless, it would be helpful to situate more explicitly the authors’ classification with respect to these other attempts in the literature. Also, they might want to reconsider their terminology, especially the characterization of 10 assignments/ $1000 as a ‘full time job’ which seems a bit too far stretched.

 Finally, I appreciate the authors’ capacity to take into account user’s concrete practices as reflected in multi-homing (simultaneous use of multiple platforms) and account sharing (more than one person working on the same account). The difficulty, as they rightly stress, is to accurately quantify them, because they have only been observed in non-representative samples. Perhaps it could be added that the question of the discrepancy between registration and real usage arises again with multi-homing, as a worker may have a different level of activity on each platform. Account-sharing also raises specific questions because it is formally forbidden on several platforms: in these cases, it may go under-reported, or perhaps it may just be limited to highly active and experienced users who have devised ways to conceal it. In passing, it is interesting to notice how smaller and even qualitative studies were necessary to retrieve this information, although the design of the paper is essentially quantitative.

 A final comment concerning the global estimate of 163M online workers: are these numbers large or small? The answer depends on the perspective taken and the goals – some will find this change huge, others will wonder why bother for such a tiny part of global production. Nevertheless, these estimates demonstrate that this population exists and needs attention – despite its limited visibility, fuzzy definition and contested boundaries. According to the authors, it has also grown rapidly. Future research will have to combine this result with further evidence on the geographical spread of this population and any effects of the COVID-19 pandemic that may have accelerated its growth.

 A minor question:

 In the Results section, it is said ‘Further adjusting for multi-homing, these numbers reduce to 10 million and 2.7 million, respectively. Finally, adjusting for possible multi-working increases these numbers to 8.5 million and 2.3 million, respectively’. The last two figures do not seem right as numbers should increase rather than decrease here.

If applicable, is the statistical analysis and its interpretation appropriate?

Yes

Is the study design appropriate and is the work technically sound?

Yes

Is the work clearly and accurately presented and does it engage with the current literature?

Yes

Are the conclusions drawn adequately supported by the results?

Yes

Are sufficient details of methods and analysis provided to allow replication by others?

Yes

Are all the source data and materials underlying the results available?

Yes

Reviewer Expertise:

Digital platform labour.

I confirm that I have read this submission and believe that I have an appropriate level of expertise to confirm that it is of an acceptable scientific standard.

## References

[1] : Ghost Work: How to Stop Silicon Valley from Building a New Global Underclass. *Boston, MA: Houghton Mifflin Harcourt* .2019;

[2] : New evidence on platform workers in Europe: Results from the second COLLEEM survey. *Publications Office of the European Union* .2020;JRC 118570: 10.2760/459278 10.2760/459278

# Response

**Published:** 2021-08-25

**Authors:** Fabian Stephany

**Affiliations:** 22137, UK

Dear reviewer,

 Thank you very much for your time and effort - the revised version of this work has benefited significantly from your comments. In summary, based on the suggestions made by all reviewers, we have corrected some calculation errors, made further clarifications on the rationale and procedure of our big data approach, and provided additional graphical evidence for the goodness of our estimations. In addition to the supplementary material of the initial version, we now provide a summary table listing all 351 platforms that are included in our analysis together with information on the type of online work performed on the platform, the number of observed (experienced) workers, and details on where this information has been retrieved. In the following, we would like to respond to your individual comments:

*"The very first step of their analysis, before any calculations, consisted in establishing a list of over 300 relevant online platforms, and gathering publicly available information about them (whether self-disclosed by the platforms or presented in other sources, e.g. the press or Wikipedia). This was done, basically, by hand, following a protocol that relies on an external, rich source (Crunchbase), a previous study of the same team, and a systematic online search. This should perhaps be recognized more explicitly, not only to give credit to the painstaking work of compiling the list, but also to discuss the issues that may have arisen – and that may add to the authors’ discussion of the uncertainties related to the calculation."*

 We have added a paragraph that recognises the platform selection process via Crunchbase in greater detail.

*"Similarly, the authors must have had to make decisions on how to deal with heterogeneity of information released by platforms…. Again, researchers have to make choices – take whatever platforms provide, approximations, or as precise figures as possible? And what happens when sources disagree?" A new table, which is provided in the data repository linked to the paper, now provides this information on all 351 platforms. "Finally, I appreciate the authors’ capacity to take into account user’s concrete practices as reflected in multi-homing…. Perhaps it could be added that the question of the discrepancy between registration and real usage arises again with multi-homing, as a worker may have a different level of activity on each platform. Account-sharing also raises specific questions because it is formally forbidden on several platforms: in these cases, it may go under-reported, or perhaps it may just be limited to highly active and experienced users who have devised ways to conceal it."*

 This is an important limitation, which has now been acknowledged in the revised version. "In the Results section, it is said ‘Further adjusting for multi-homing, these numbers reduce to 10 million and 2.7 million, respectively. Finally, adjusting for possible multi-working increases these numbers to 8.5 million and 2.3 million, respectively’. The last two figures do not seem right as numbers should increase rather than decrease here." This mistake has now been corrected.
